# Detoxification of hydrogen sulfide by synthetic heme model compounds

**DOI:** 10.1038/s41598-024-80511-1

**Published:** 2024-12-10

**Authors:** Atsuki Nakagami, Qiyue Mao, Masaki Horitani, Masahito Kodera, Hiroaki Kitagishi

**Affiliations:** 1 Department of Molecular Chemistry and Biochemistry, Faculty of Science and Engineering, Doshisha University, 1-3 Tatara Miyakodani, Kyotanabe-city, Kyoto 610-0321 Japan; 2 Department of Applied Biochemistry and Food Science, Faculty of Agriculture, Saga University, 1 Honjo-machi, Saga, 840-8502 Japan; 3 The United Graduate School of Agricultural Science, Kagoshima University, 1-21-24 Korimoto, Kagoshima, 890-0065 Japan

**Keywords:** Hydrogen sulfide, Heme, Porphyrin, Cyclodextrin, Injectable antidote, Bioinorganic chemistry, Medicinal chemistry, Toxicology, Supramolecular chemistry, Translational research

## Abstract

Hydrogen sulfide is a lethal toxic gas that disrupts cellular respiration in the mitochondrial system. Currently, no antidote is available for the clinical treatment of hydrogen sulfide poisoning. In this study, we investigated the function of iron(III)porphyrin complexes as hydrogen sulfide scavengers in water and evaluated their potential use as therapeutic agents for hydrogen sulfide poisoning. The compounds, named met-hemoCD-P and met-hemoCD-I, are composed of iron(III)porphyrin complexed with per-methylated β-cyclodextrin dimers that contain a pyridine (met-hemoCD-P) or imidazole axial fifth ligand that is coordinated to Fe(III) (met-hemoCD-I). These compounds formed stable HS–Fe(III) complexes under physiological conditions, with binding constants of 1.2 × 10^5^ and 2.5 × 10^6^ M^–1^ for met-hemoCD-P and met-hemoCD-I, respectively. The binding constant of met-hemoCD-I was 10-times higher than that reported for native human met-hemoglobin at pH 7.4 and 25^o^C. Electron paramagnetic resonance (EPR) spectroscopy and H_2_S quantification assays revealed that after SH^–^ was coordinated to met-hemoCD-I, it was efficiently converted to nontoxic sulfite and sulfate ions via homolytic cleavage of the HS–Fe(III) bond followed by aerobic oxidation. Mouse animal experiments revealed that the survival rate was significantly improved when NaSH-treated mice were injected with met-hemoCD-I. After the injection, mitochondrial C*c*O function in brain and heart tissues recovered, and met-hemoCD-I injected was excreted in the urine without chemical decomposition.

**Supplementary Information:**

The online version contains supplementary material available at 10.1038/s41598-024-80511-1.

## Introduction

Hydrogen sulfide (H_2_S) is a colorless, flammable, and hazardous gas with a rotten egg smell. The toxic effect of H_2_S is similar to that of hydrogen cyanide (HCN), which strongly binds to cytochrome *c* oxidase (C*c*O) in the mitochondrial respiratory chain; thus, H_2_S is classified as a cellular asphyxiant^1–5^. As its specific gravity (1.19) is greater than that of air, H_2_S tends to accumulate at lower altitudes, often causing poisoning accidents at sites such as in manholes, sewage systems, and mining operations^1,6^. Although there are fewer than 10 cases of industrial H_2_S poisoning per year in Japan, 220 cases with 208 deaths were reported in 2007 in Japan due to suicide by intentional H_2_S generation, which is known as the detergent suicide pandemic^6,7^. The following year, this method of suicide was observed in the United States and worldwide^8,9^. Frighteningly, residual H_2_S gas often spreads from the source or victim and causes secondary poisoning to the rescue personnel and/or individuals at the site^8,9^. No clinical antidote is currently available for H_2_S poisoning. Therefore, ready-to-use antidotes that can be stored for long durations and are immediately effective are greatly needed, especially for situations involving emergency rescue.

**Fig. 1:**
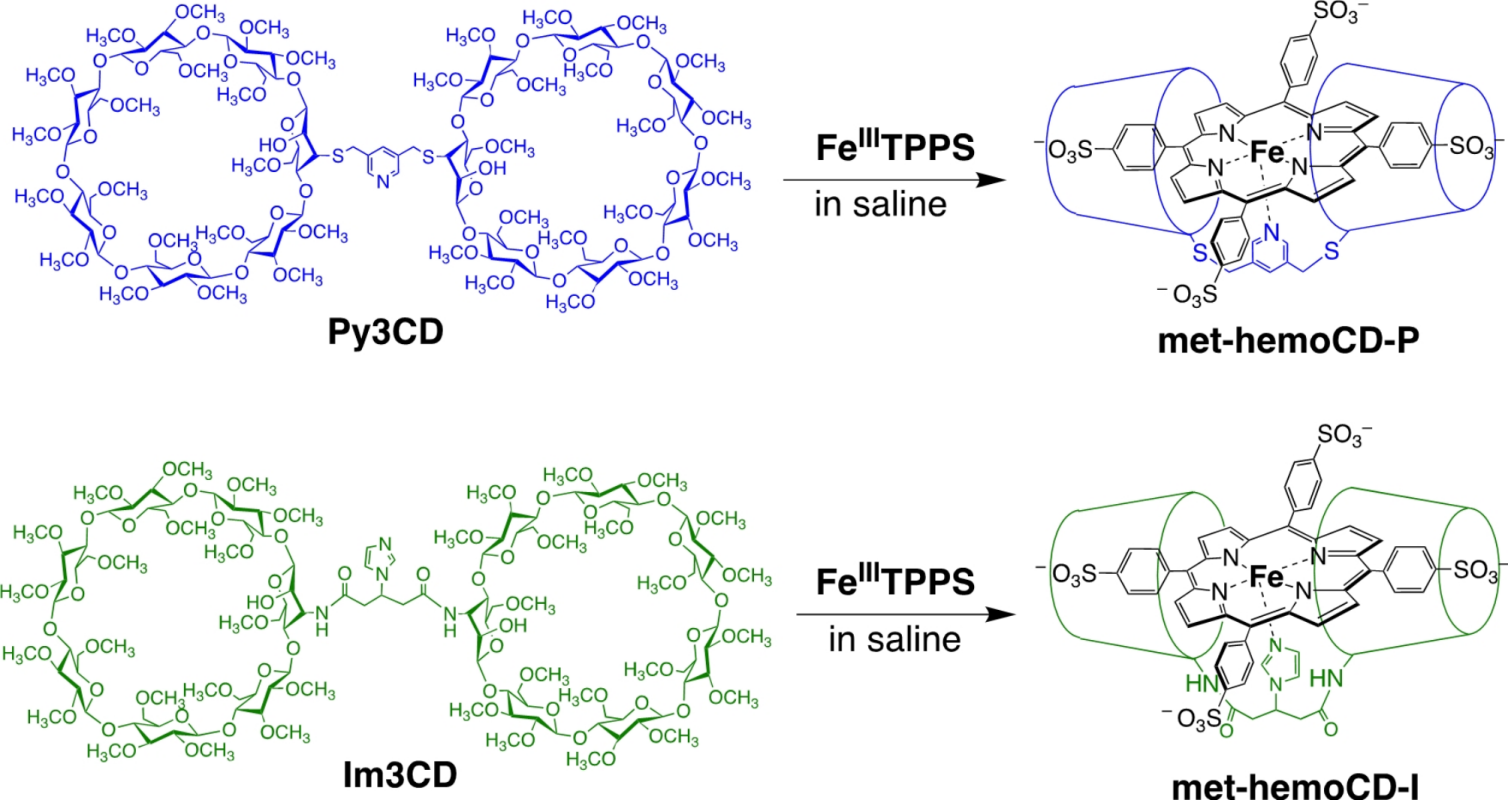
Chemical structures of cyclodextrin dimers Py3CD and Im3CD and met-hemoCD-P and met-hemoCD-I in combination with iron(III)porphyrin (Fe(III)TPPS) in aqueous saline solution.

The administration of heme proteins or artificial heme-model compounds may be a promising approach for the development of antidotes against poisoning caused by inhaled gases such as H_2_S, which reacts with metalloproteins (including hemoglobin) in red blood cells (RBCs)^10,11^. For this purpose, the compounds should exhibit higher binding affinities toward toxins than native hemes. Relying on this strategy, researchers have proposed potential antidotes for carbon monoxide (CO), hydrogen cyanide (HCN), and H_2_S poisoning using native and modified heme proteins as well as natural vitamin B_12_ analogs^4,12–18^. However, few studies have established antidote systems using synthetic compounds^12,19^. Therefore, our group developed synthetic heme-model compounds composed of iron tetrakis(4-sulfonatophenyl)porphyrin (FeTPPS) complexes encapsulated by per-*O*-methylated β-cyclodextrin (CD) dimers^20–22^. Figure 1 shows two representative CD dimers, Py3CD and Im3CD, that form inclusion complexes with Fe(III)TPPS to yield met-hemoCD-P and met-hemoCD-I, respectively. We have shown that reduced hemoCD-P in the ferrous state functions as an internal CO scavenger in vivo^21,23^ and that met-hemoCD-I functions as a potential cyanide antidote^24,25^. When these heme model compounds were injected intravenously or intraperitoneally into mice or rats, they bound gaseous molecules in the circulation system^21–25^. Interestingly, these compounds were rapidly and quantitatively excreted in the urine through renal clearance; thus, they do not accumulate in the body. Therefore, compared to native protein-based scavenging systems, our system shows potential as an injectable antidote.

In the present study, we investigated the potential of met-hemoCD-P and met-hemoCD-I as hydrogen sulfide scavengers. Using the ferric forms of these two complexes, we first present the basic reactivity toward hydrogen sulfide in aqueous solution in view of thermodynamic and kinetic parameters and spectroscopic characterizations. Then, the antidote effect against hydrogen sulfide-induced intoxication was tested in mice.

## Results

### Binding of hydrogen sulfide to met-hemoCD-P and met-hemoCD-I

**Fig. 2:**
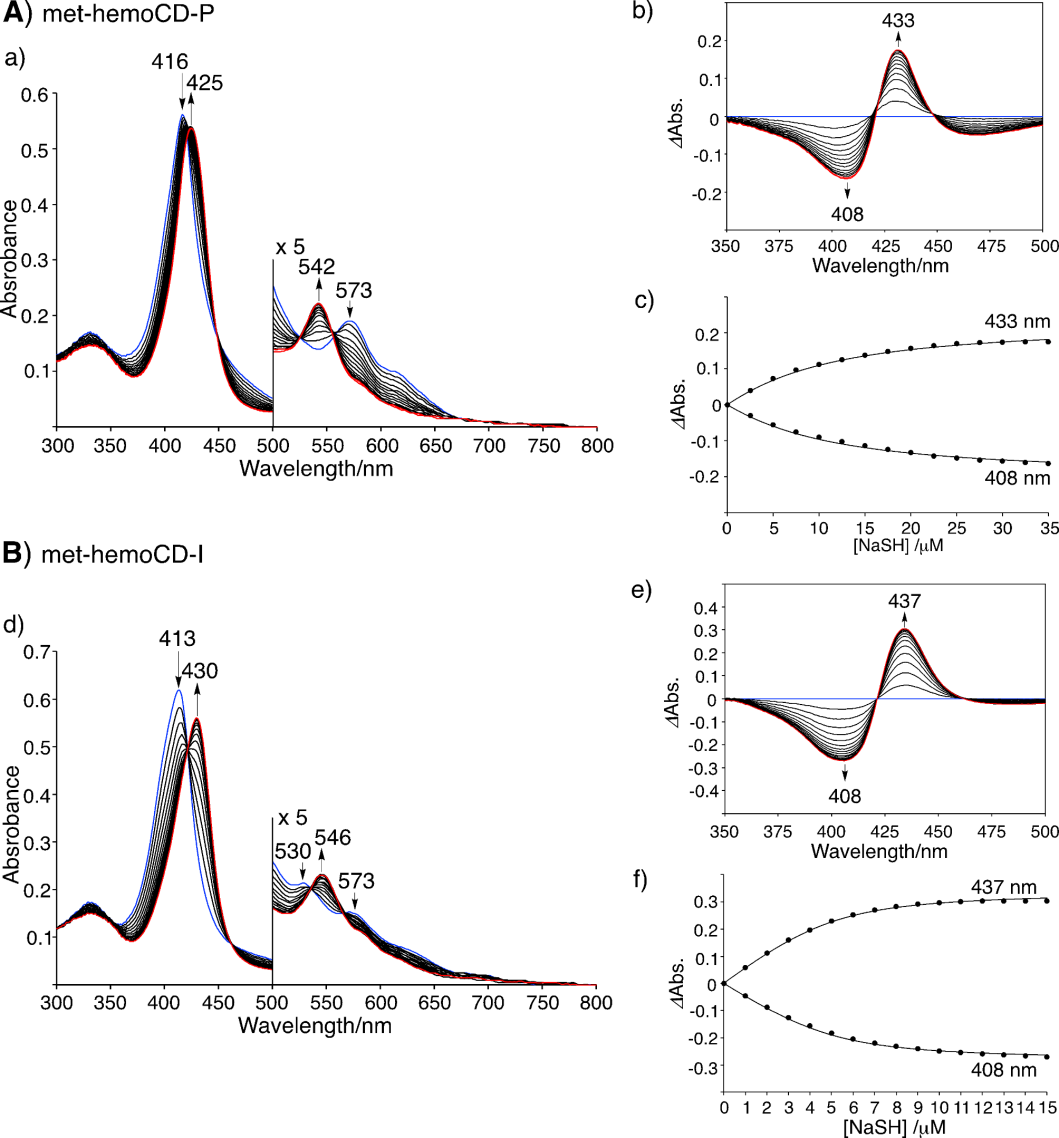
Spectroscopic analysis of the binding of hydrogen sulfide to (**A**) met-hemoCD-P (5 µM) and (**B**) met-hemoCD-I (5 µM) in 0.05 M phosphate buffer solution at pH 7.4 and 25 °C. Changes in the UV‒vis absorption spectra of (a) met-hemoCD-P and (d) met-hemoCD-I upon the addition of NaSH. The initial spectrum is shown in blue, and the final spectrum is shown in red. The differential spectra of (b) met-hemoCD-P and (e) met-hemoCD-I upon the addition of NaSH. The plots of the absorbance changes versus [NaSH] of (c) met-hemoCD-P and (f) met-hemoCD-I. The solid lines are the best fit of the data to an equation for 1:1 complex formation to give the binding constants (*K*).

In this study, we used sodium hydrogen sulfide (NaSH) as the sulfide source. In aqueous media, SH^–^ equilibrates with H_2_S, and the p*K*_a_ is 6.98 at 25 °C and 6.76 at 37 °C^26,27^. The presence of S^2–^ could be negligible under aqueous conditions due to the low acidity of SH^–^ (p*K*_a_ = 19 ± 2).^26,27^ In this article, we use the term “hydrogen sulfide” to refer to both H_2_S and SH^–^ species. The binding constants (*K*) of hydrogen sulfide to met-hemoCD-I and met-hemoCD-P were evaluated by UV‒vis spectroscopic titration. Upon the addition of NaSH, the spectra of met-hemoCD-P and met-hemoCD-I changed stepwise with clear isosbestic points (Fig. 2). The spectral changes were saturated at approximately one equivalent of added NaSH. The spectral characters of these complexes were shown in Figures S1 and S2. In the absence of CD dimers, Fe(III)TPPS was decomposed by the addition of NaSH (Figure S3A)^28,29,45^. Similar porphyrin decomposition was also observed for Fe(III)TPPS complexed with 2,3,6-tri-*O*-methyl-β-CD (TMe-β-CD), which lacks an axial fifth ligand (Figure S3B). This result suggested that, in addition to protection by the CD cavity, pyridine or imidazole ligation in Py3CD or Im3CD contributed to the formation of a stable HS–Fe(III)porphyrin complex.

**Fig. 3:**
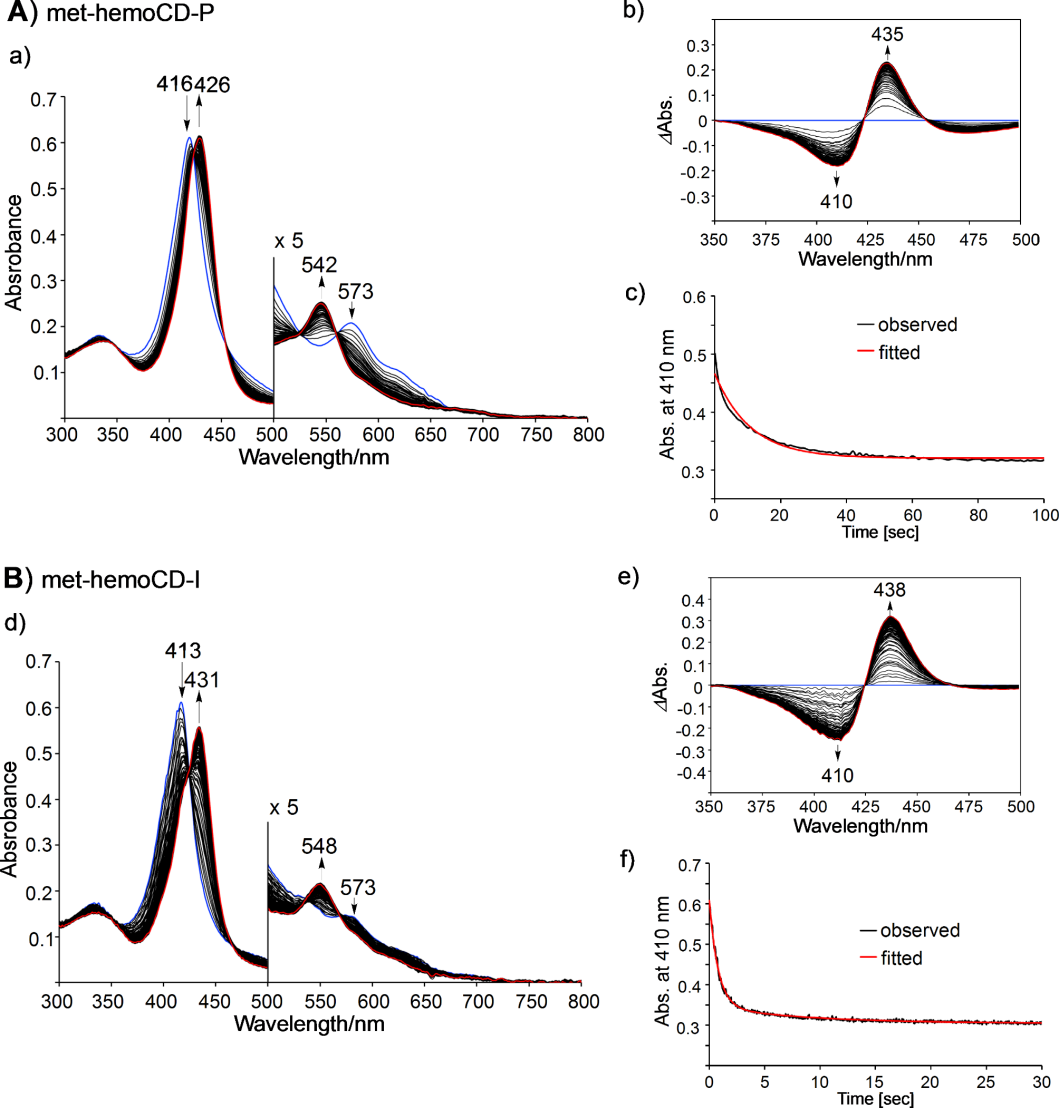
UV‒vis absorption spectra collected over time in the reaction of (**A**)met-hemoCD-P (5 µM) and (**B**) met-hemoCD-I (5 µM) with excess NaSH in 0.05 M phosphate buffer solution at pH 7.4 and 25 °C. The initial spectrum is shown in blue, and the final spectrum is shown in red. The differential spectra of (b) met-hemoCD-P and (e) met-hemoCD-I upon the addition of NaSH. The time-course traces at 410 nm (black) and the fitted curve (red) according to a single/double exponential function to derive the observed rate constants (*k*_obs_) for (c) met-hemoCD-P and (f) met-hemoCD-I.

**Fig. 4:**
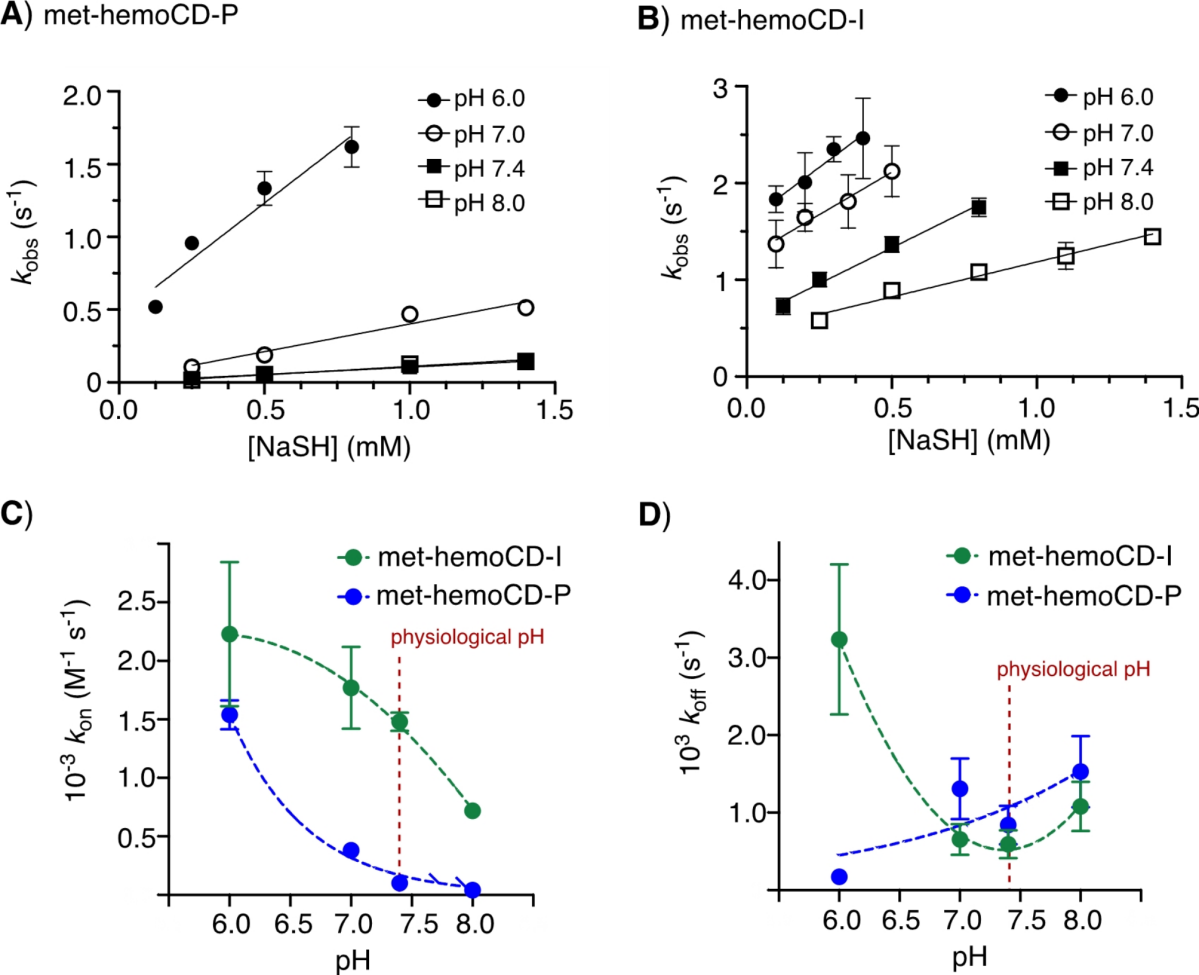
Kinetic analysis of the binding between hydrogen sulfide and met-hemoCDs. (**A**,**B**) Plots of *k*_obs_ for met-hemoCD-P and met-hemoCD-I as a function of [NaSH]. The solid lines represent linear least-square fitting of the data, which provided the *k*_on_ values. (**C**,**D**) Plots of *k*_on_ and *k*_off_ for met-hemoCD-P and met-hemoCD-I as a function of pH.

The titration curves were well fitted to the 1:1 equilibrium model, affording *K* values of 1.2 × 10^5^ M^–1^ and 2.5 × 10^6^ M^–1^ for met-hemoCD-P and met-hemoCD-I, respectively, in phosphate buffer at pH 7.4 (Fig. 2). The kinetic parameters for the binding of hydrogen sulfide were determined by time-resolved UV‒vis spectral measurements (Fig. 3). Single/double-exponential curve fitting analysis was used to determine the apparent association rate constants (*k*_on_^app^, the fast component in the double-exponential fitting analysis (> 80%)). The second-order rate constants (*k*_on_) were determined via linear regression of the *k*_on_^app^ values as a function of [NaSH] (Fig. 4A and B). The parameters were strongly dependent on the pH of the solution (Figure S4 and S5). As listed in Table 1, met-hemoCD-I exhibits higher *K* and *k*_on_ values than those of met-hemoCD-P. The plot of *k*_on_ and *k*_off_ values versus pH (Fig. 4C and D) clearly shows the high ability of met-hemoCD-I to act as a hydrogen sulfide scavenger at physiological pH (7.4). Hydrogen sulfide binds more quickly to met-hemoCD-I at neutral pH because the p*K*_a_ (p*K*_a_^H2O^ = 7.7) of the axial aqua ligand is higher than that of hemoCD-P (p*K*_a_^H2O^ = 5.5).^22,24^

**Table 1:** Binding constants (*K*) and rate constants (*k*_on_, *k*_off_) of met-hemoCD-P and met-hemoCD-I with hydrogen sulfide in 0.05 M phosphate buffer at different pH values and at 25 °C.

	met-hemoCD-*P*			met-hemoCD-I		
pH	*K* (M^–1^)	*k*_on_ (M^–1^s^–1^)	*k*_off_ (s^–1^)	*K* (M^–1^)	*k*_on_ (M^–1^s^–1^)	*k*_off_ (s^–1^)
6.0	(9.0 ± 2.6) x 10^6^	(1.5 ± 0.1) x 10^3^	(1.7 ± 1.0) x 10^–4^	(6.9 ± 2.0) x 10^5^	(2.2 ± 0.6) x 10^3^	(3.2 ± 1.0) x 10^–3^
7.0	(2.9 ± 1.0) x 10^5^	(3.8 ± 0.3) x 10^2^	(1.3 ± 0.4) x 10^–3^	(2.7 ± 0.8) x 10^6^	(1.8 ± 0.4) x 10^3^	(6.6 ± 2.0) x 10^–4^
7.4	(1.2 ± 0.4) x 10^5^	(1.0 ± 0.1) x 10^2^	(8.4 ± 3.0) x 10^–4^	(2.5 ± 0.7) x 10^6^	(1.5 ± 0.8) x 10^3^	(5.9 ± 1.0) x 10^–4^
8.0	(3.0 ± 1.0) x 10^4^	(4.0 ± 1.0) x 10^1^	(1.5 ± 0.5) x 10^–3^	(6.7 ± 2.0) x 10^5^	(7.2 ± 4.4) x 10^2^	(1.1 ± 0.3) x 10^–3^

**Table 2:** Binding constants (*K*) and rate constants (*k*_on_, *k*_off_) of met-hemoCD, met-Hb and met-Mb with hydrogen sulfide in aqueous buffer at pH 7.4 and 25 °C.

	K (M^–1^)	k_on_ (M^–1^s^–1^)	k_off_ (s^–1^)
met-hemoCD-P	1.2 × 10^5^	1.0 × 10^2^	8.4 × 10^–4^
met-hemoCD-I	2.5 × 10^6^	1.5 × 10^3^	5.9 × 10^–4^
met-Mb (horse)^*a,d,f*^	1.0 × 10^4^	1.6 × 10^4^	1.6
met-Hb (human)^*b,c,f*^	2.8 × 10^5^	9.9 × 10^2^	3.5 × 10^–3^
met-Mb (sperm whale)^*b*,*c,e,f*^	9.6 × 10^4^	4.6 × 10^3^	4.8 × 10^–2^

^*a*^Ref. 37.

^*b*^Ref. 38.

^*c*^The kinetic parameters were recalculated as a function of the hydrogen sulfide (H_2_S/SH^–^) concentration.

^*d*^Data obtained at 37 °C.

^*e*^Data obtained at 20 °C.

^*f*^Na_2_S was used as a sulfide source.

A continuous variation plot (Job plot) was constructed for the met-hemoCD-I and NaSH systems (Figure S6). The maximum complexation ratio observed at a 1:1 molar ratio clearly indicates that a 1:1 complex of met-hemoCD-I and NaSH was present; these results indicate that poly(sulfide) complexes such as HS(S)_n_–Fe(II), which are often proposed in biological systems, were not detected^11^. Additionally, electrospray ionization time-of-flight mass spectrometry (ESI-TOF-MS) revealed that the HS–Fe(III) complex in met-hemoCD-I formed (Figure S7). The molecular ion peak of met-hemoCD-I (MW = 3947.9) was detected at 1315.4 (*m*/*z*) as a tri-anionic species. With NaSH, the molecular ion peak of met-hemoCD-I was mainly detected at 995.1 (*m*/*z*), which could be assigned to HS-bound met-hemoCD-I (MW = 3981.0) in the tetra-anionic form. These results revealed that a stable 1:1 complex of met-hemoCD-I with hydrogen sulfide (HS–Fe(III)porphyrin) formed in aqueous media at physiological pH.

### Oxidative degradation of hydrogen sulfide by met-hemoCD-I

**Fig. 5:**
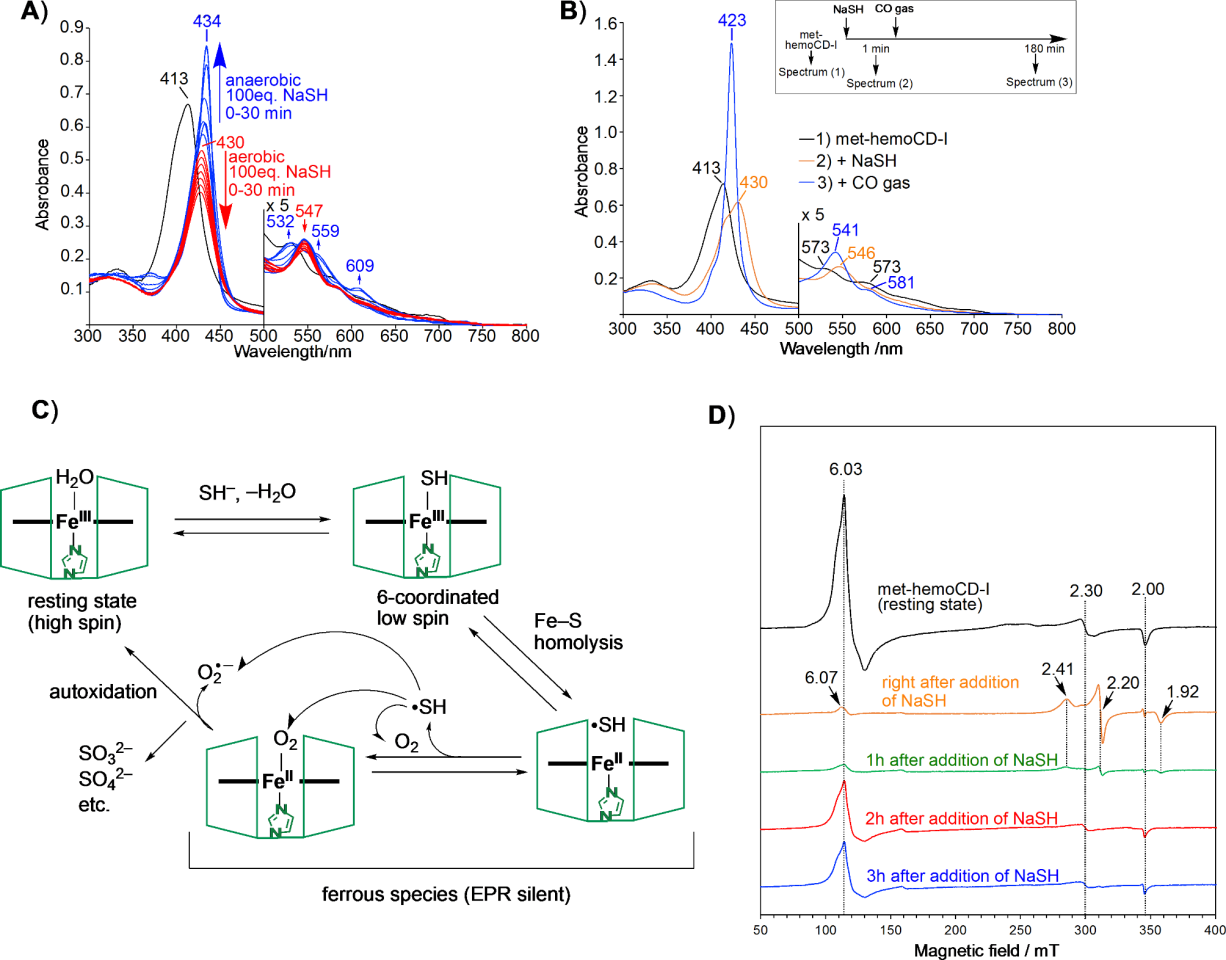
Reaction of met-hemoCD-I with hydrogen sulfide in 0.05 M phosphate buffer solution at pH 7.4. (**A**) Changes in the UV‒vis absorption spectra of met-hemoCD-I (5 µM) (black line) after the addition of excess NaSH under aerobic (red lines) and anaerobic (blue lines) conditions at 25 °C. (**B**) UV‒vis absorption spectra of met-hemoCD-I (5 µM) before and after two molar equivalents of NaSH were added at 25 °C. After the formation of HS-hemoCD-I complex, the solution was bubbled with CO gas right after the CO-ferrous complexes were formed. (**C**) Proposed pathway for the catalytic decomposition of hydrogen sulfide with met-hemoCD-I. (**D**) EPR spectral changes of met-hemoCD-I before and after NaSH (two equivalents) was added. The reagents were mixed and incubated at 25 °C, and the spectra were measured in the frozen state at 5 K.

In the presence of excess NaSH (100 equivalents), met-hemoCD-I gradually degraded under aerobic conditions (Fig. 5A and S8). The degradation was significantly suppressed under anaerobic conditions with a Soret band at 434 nm, indicating that a ferrous hemoCD-I complex formed in the deoxy form^30^. To confirm the iron oxidation state, carbon monoxide (CO) gas was introduced into the solution after met-hemoCD-I was mixed with NaSH (Fig. 5B, S9 and S10). A sharp Soret band appeared at 423 nm, which is characteristic of the CO–Fe(II) complex. Therefore, homolytic bond cleavage of the HS–Fe(III) complex occurred, generating a sulfide radical (HS•) and Fe(II) complex of hemoCD-I. In the presence of molecular oxygen (O_2_), the reduced Fe(II) complex formed the O_2_ adduct, which was readily autoxidized to ferric met-hemoCD-I with the generation of superoxide^31–33^. The autoxidation rate of the O_2_ adduct for hemoCD-I (*t*_1/2_ ~ 36 min at 37 °C) is much faster than that for ferrous hemoCD-P (*t*_1/2_ ~ 5 h at 37 °C)^25^. Therefore, the reaction cycle of met-hemoCD-I with hydrogen sulfide under aerobic conditions should proceed faster than met-hemoCD-P. In addition, as the autoxidation of oxy-hemoCD-I became fast in the presence of SH^–^ (Figure S11), there are multiple pathways of sulfide consumption in this system as the overall scheme is summarized in Fig. 5C.

To further characterize the reaction of met-hemoCD-I with NaSH, electron paramagnetic resonance (EPR) spectra of met-hemoCD-I were obtained before and after the reaction of NaSH at physiological pH and 5 K (Fig. 5D). Before the reaction with NaSH occurred, met-hemoCD-I showed EPR signals at *g* = 6.03, 2.30, and 2.00. The signals at *g* = 6.03 and 2.00 could be assigned to the characteristic signals of 5-coordinated high-spin iron(III)porphyrin with or without a weakly coordinated sixth ligand, such as H_2_O, while the signal at *g* = 2.30 could be assigned to the iron(III)porphyrin coordinated with a hydroxoligand^34^. Immediately after NaSH was added, characteristic signals at *g* = 2.41, 2.20, and 1.92 were generated due to the HS–Fe(III)–N(imidazole) 6-coordinated low-spin complex^35–37^. The EPR signals became almost silent when the solution was incubated for an hour and then frozen after NaSH was added, indicating that an EPR-inactive ferrous Fe(II) complex formed due to the homolysis of HS–Fe(III). Ferric high-spin species of met-hemoCD-I were detected after 2 and 3 h of incubation. The time-course change in the EPR spectra confirmed that met-hemoCD-I was converted to the ferrous Fe(II) complex via HS–Fe(III) complex formation, as proposed in Fig. 5C.

**Fig. 6:**
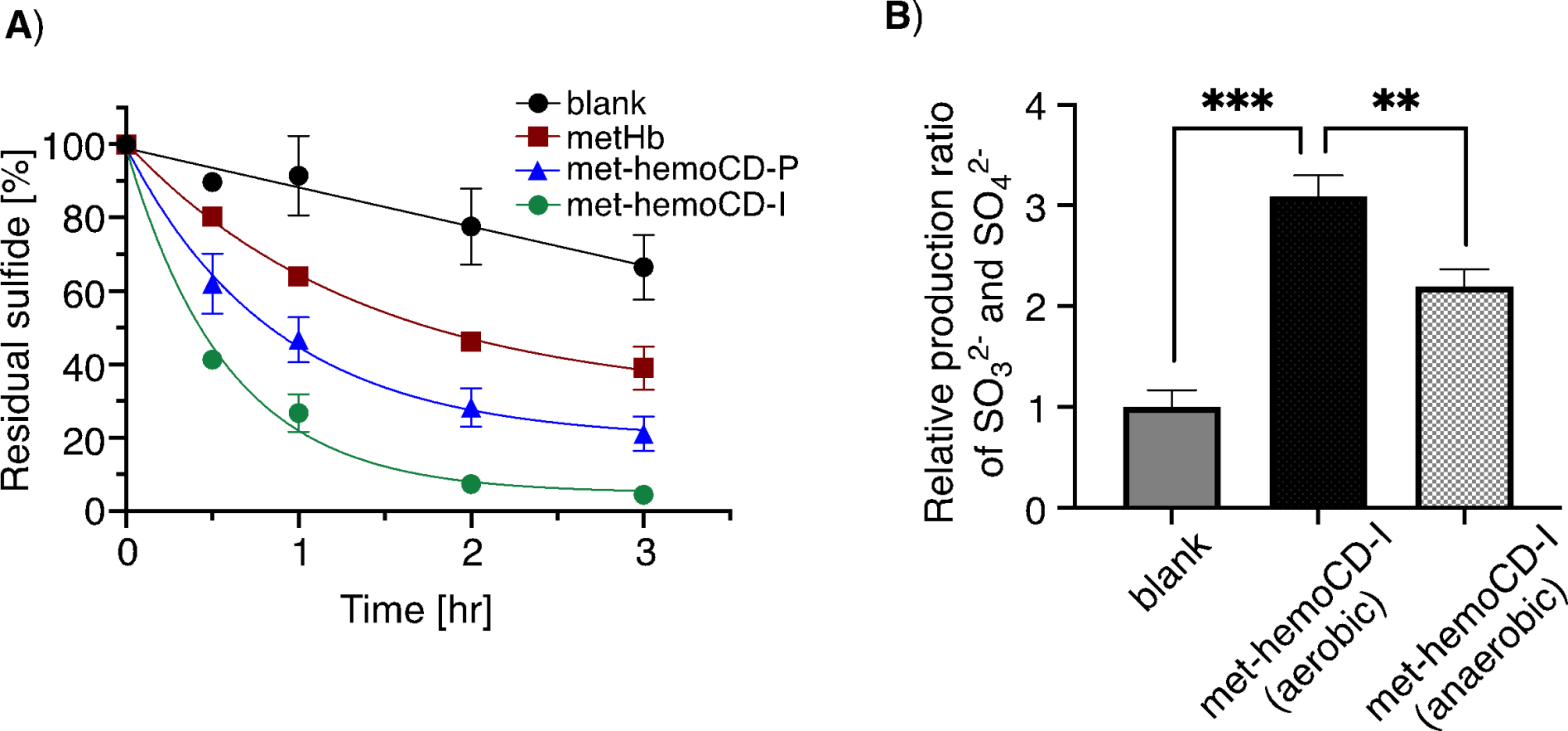
Degradation of hydrogen sulfide in 0.05 M phosphate buffer solution at pH 7.4. (**A**) Time course changes in the residual amount of hydrogen sulfide (initial: 1.0 mM) in solutions containing met-hemoCD-I, met-hemoCD-P, and met-Hb (50 µM each). The residual sulfide was detected by the methylene blue method (see Materials and Methods section). (**B**) Production of sulfite and sulfate ions during the reaction between hydrogen sulfide and met-hemoCD-I. The aqueous solution of hydrogen sulfide (9 mM) was mixed with met-hemoCD-I (0.45 mM) and incubated for 1 h. The sulfite and sulfate ions were detected by the addition of barium chloride and quantified by the solution turbidity (see Experimental section). Each bar represents the mean ± SE (*n* = 3). The asterisks denote statistical significance, ***P < 0.005. **P < 0.01.

The hydrogen sulfide species in aqueous solution decomposed rapidly in the presence of met-hemoCD-I (Fig. 6A). The efficacy of hydrogen sulfide decomposition is correlated with binding parameters for met-hemoCD-I, met-hemoCD-P, and met-Hb, as summarized in Table 2. Consistently, sulfite and sulfate ions were produced efficiently in the presence of O_2_ with met-hemoCD-I (Fig. 6B). The control data under anaerobic conditions support the involvement of O_2_ in met-hemoCD-I-assisted decomposition of hydrogen sulfide. Other sulfide compounds such as polysulfides and thiosulfate could be produced as previously reported^37^ but were not quantified in this study. Turbidimetric assay using barium ions supports the formation of BaSO_3_ and BaSO_4_ as the precipitates. The significant production of sulfite and sulfate ions in the presence of met-hemoCD-I under anaerobic conditions would be derived from high affinity of hemoCD-I against O_2_. The *P*_1/2_^O2^ value of 1.7 Torr for hemoCD-I is much smaller than that of hemoCD-P (10 Torr)^22^. A small amount of residual oxygen could bind to met-hemoCD-I, which causes production of these anions even under anaerobic conditions.

### Strength of met-hemoCD-I antidotes for hydrogen sulfide poisoning in mice

**Fig. 7:**
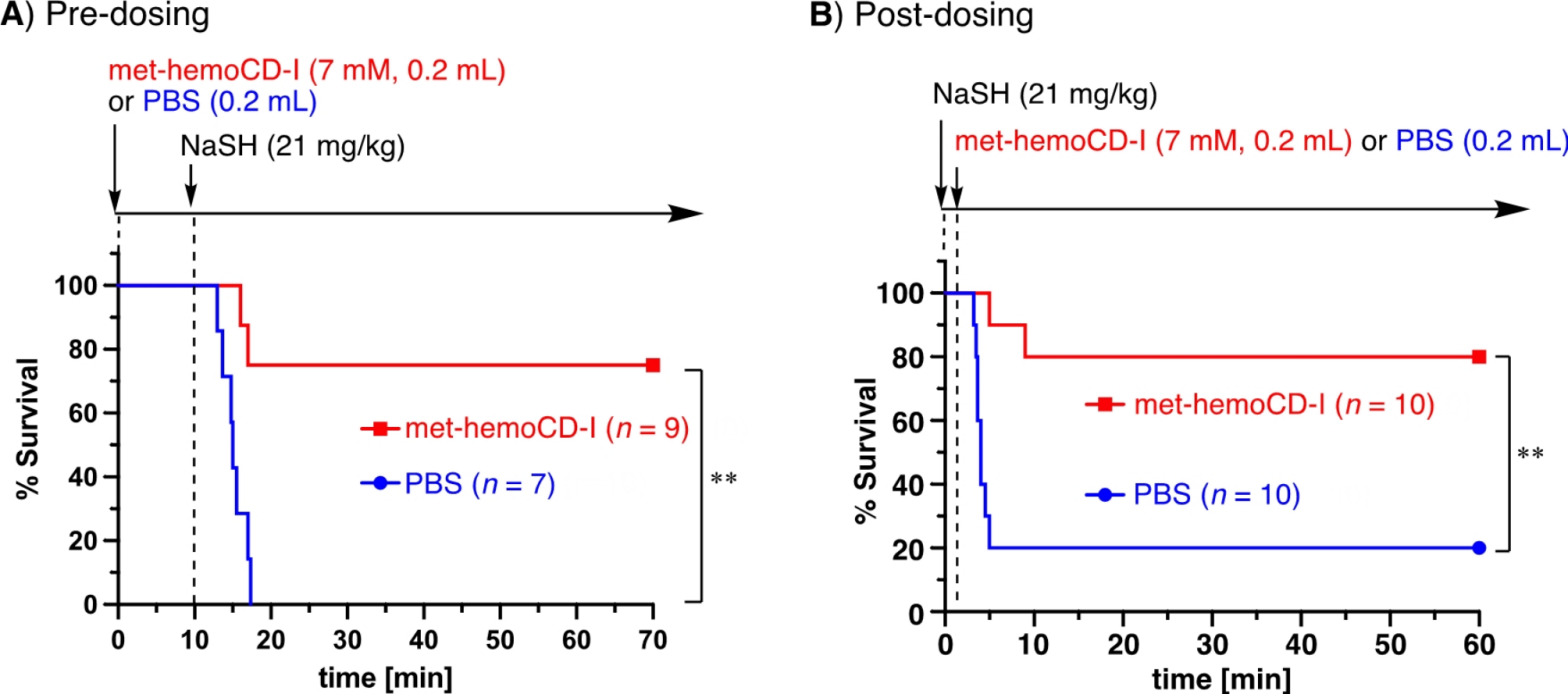
Survival curves for NaSH-treated mice treated with or without met-hemoCD-I. (**A**) Predosing. A solution of met-hemoCD-I (7 mM, 0.2 mL) in PBS was intraperitoneally injected into the mice, followed by an intraperitoneal injection of NaSH (21 mg/kg). (**B**) Postdosing. A solution of NaSH (21 mg/kg) in PBS was intraperitoneally injected into the mice, followed by an intraperitoneal injection of met-hemoCD-I (7 mM, 0.2 mL). The asterisks denote statistical significance, **P < 0.01.

We then evaluated the strength of met-hemoCD-I antidotes for hydrogen sulfide in mice. As shown in the survival curve of the mice in Fig. 7A, the intraperitoneal injection of NaSH (21 mg/kg) into the mice caused significant lethal toxicity. When 7 mM met-hemoCD-I aqueous solution (0.2 mL) was intraperitoneally injected prior to NaSH injection, the survival curve significantly improved, indicating the efficacy of NaSH detoxification. The antidote effect was also significant when met-hemoCD-I was injected immediately after the mice were poisoned with NaSH (Fig. 7B). Therefore, met-hemoCD-I is effective before and after poisoning with NaSH.

**Fig. 8:**
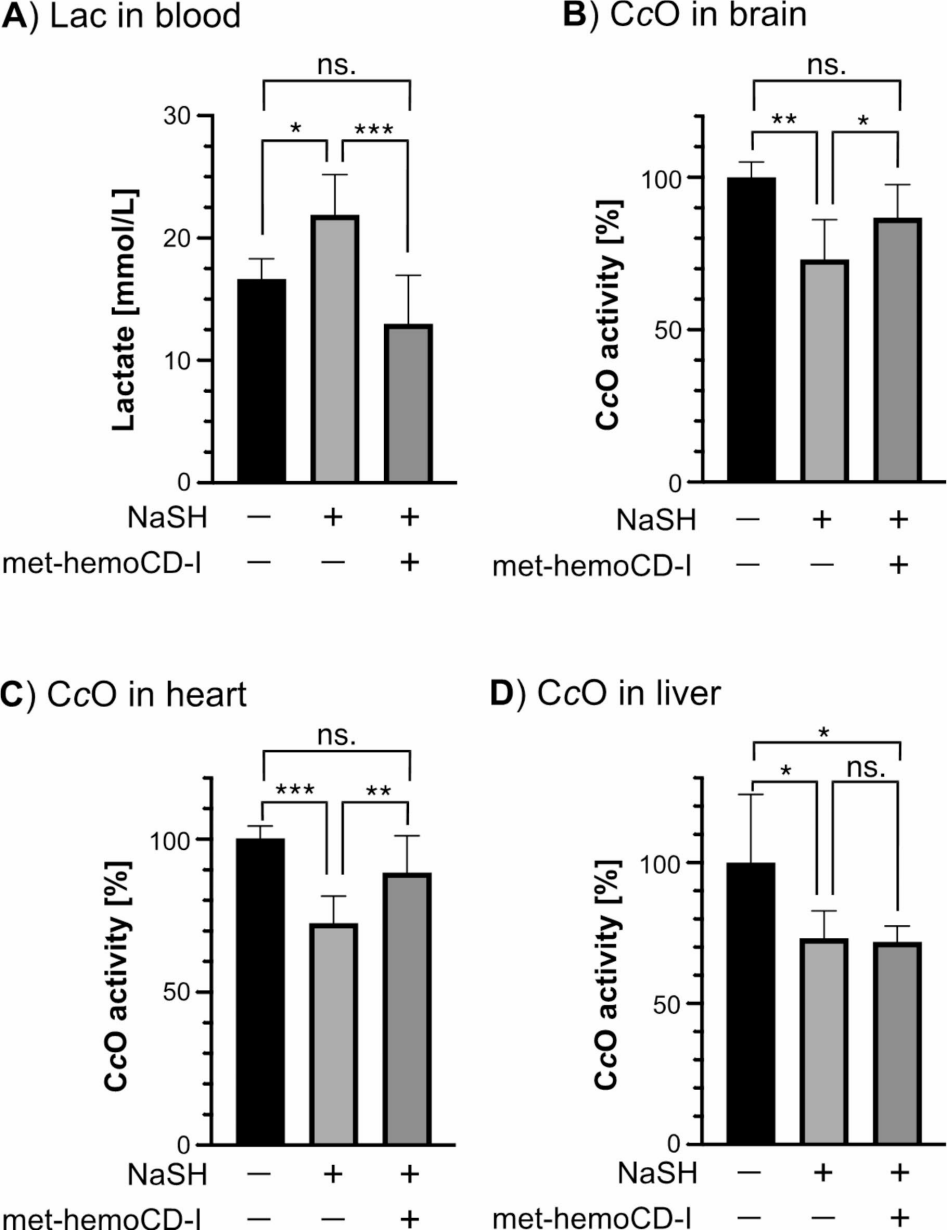
Effect of met-hemoCD-I antidotes on mice poisoned by NaSH. (**A**) Lactate levels in the blood of mice treated with NaSH followed by met-hemoCD-I. (**B**–**D**) Cytochrome *c* oxidase (C*c*O) activity in the brain (**B**), heart (**C**), and liver (**D**) of mice treated with NaSH followed by met-hemoCD-I. In the model, a solution of NaSH (21 mg/kg) in PBS was intraperitoneally injected into mice, followed by an intraperitoneal injection of met-hemoCD-I (7 mM, 0.2 mL). Each bar represents the mean ± SE (*n* ≧ 3). The asterisks denote statistical significance, ***P < 0.005. **P < 0.01, *P < 0.05.

The lactate level in the blood was increased by NaSH but returned to normal by met-hemoCD-I (Fig. 8A). Therefore, anaerobic metabolism caused by NaSH was recovered in mice by the injection of met-hemoCD-I. More directly, we investigated the activity of C*c*O in organs (Fig. 8B–D). In brain and heart tissues, the NaSH-induced decrease in C*c*O activity returned to normal in the met-hemoCD-I-treated mice, whereas no significant change was observed in the liver. Overall, these analyses support that met-hemoCD-I injection protects against hydrogen sulfide-induced asphyxial death in mice.

**Fig. 9:**
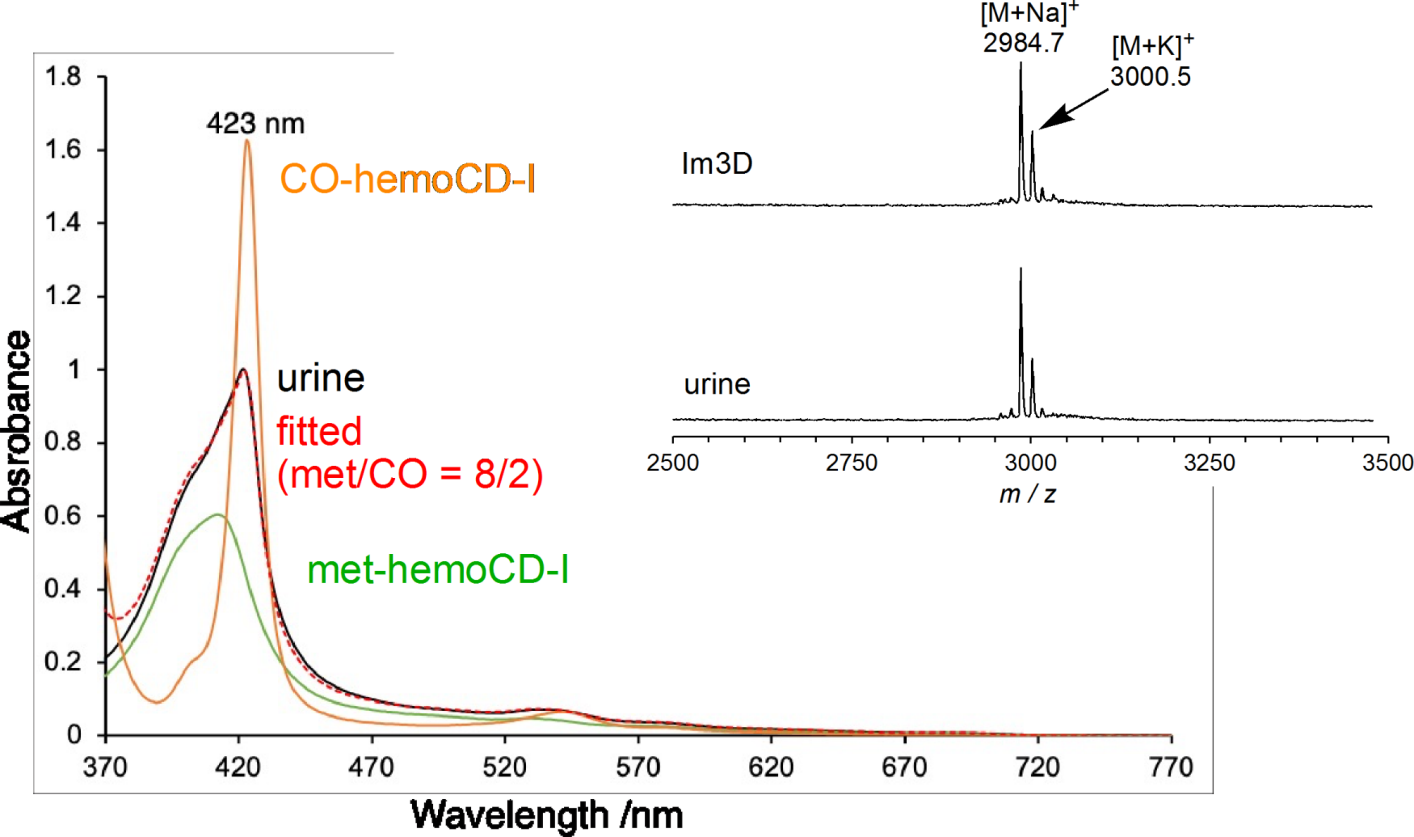
UV‒vis absorption spectrum of urine (black) excreted from mice treated with NaSH (21 mg/kg) followed by met-hemoCD-I (7 mM, 0.2 mL). The spectra of met-hemoCD-I (green) and CO-hemoCD-I (orange) are shown for comparison. The red dotted line shows the accumulated spectra of met-hemoCD-I (80%) and CO-hemoCD-I (20%), which were consistent with those of urine. The inset shows MALDI-TOF mass spectra of Im3CD and urine with subsequent addition of α-cyano-4-hydroxycinamic acid (positive mode). The calculated molecular weight (M) of Im3CD is 2961.4.

After met-hemoCD-I was injected, dark red urine was obtained from the mice within 60 min. The UV‒vis spectrum of the urine showed the characteristic Soret and Q bands of met-hemoCD-I (Fig. 9). The spectral simulation revealed that the urine contained 80% met-hemoCD-I and 20% CO-hemoCD-I. Ferrous hemoCD-I could be formed by a natural reduction system in the body^39^ and/or the homolytic cleavage of the HS–Fe(III) bond in hemoCD-I formed during circulation. Ferrous hemoCD-I bound to endogenous CO in the circulation and was subsequently excreted. Matrix-assisted laser desorption/ionization time-of-flight (MALDI-TOF) mass spectroscopic analysis of urine revealed no changes in the Im3CD structure (Fig. 9 inset). The inclusion complex of met-hemoCD-I was dissociated upon laser irradiation according to MALDI-TOF mass spectrometry. These results indicate that injected met-hemoCD-I in mice was excreted in the urine without chemical changes. Injected met-hemoCD-I could react with hydrogen sulfide and metabolize the compound to sulfite and/or sulfate during circulation, then return to the met-form via the mechanism proposed in Fig. 5C.

## Discussion

In this work, we investigated the use of met-hemoCD-P and met-hemoCD-I as hydrogen sulfide receptors in aqueous solution and in vivo. Compared to native Hb and Mb, met-hemoCD-I showed a greater binding rate and affinity toward hydrogen sulfide; therefore, we concluded that met-hemoCD-I is an effective and ready-to-use antidote for hydrogen sulfide-induced poisoning. Here, we discuss the binding of hydrogen sulfide to our heme-model system and compare it with that of other potential antidotes.

The binding of hydrogen sulfide to metal ions in proteins, including heme proteins, has been proposed in many studies^10,11^. However, compared to that of O_2_, CO, and cyanide, detailed characterizations of HS–metal complexes with thermodynamic and kinetic parameters have been limited in the several literatures^40^. Among the native systems, the interactions between ferric Hb and Mb with hydrogen sulfide have been characterized in detail^37,38^. As reported in these studies, the binding parameters strongly depend on the pH of the solutions. The binding is relatively fast at low pH, and under these conditions, hydrogen sulfide presents as H_2_S. Interestingly, the pH dependency is reversed for a small heme protein, MP-11^41^, in which the heme cofactor is exposed to the aqueous bulk phase. These data indicate that the preferable attacking species, H_2_S or SH^–^, should differ depending on the environment around the iron center of the heme^42^. In the hemoCD system, the binding parameters were also dependent on pH, similar to those of Hb and Mb, indicating that hydrophobic H_2_S tends to enter the iron(III) site located in the CD cavity. In contrast, we confirmed that a stable 1:1 complex of iron(III)porphyrin formed with SH^–^. The net charge of the iron(III)porphyrin ring becomes zero upon SH^–^ ligation (four negative charges of peripheral sulfonate locate outside the cyclodextrin’s cavity).The electronically neutralized porphyrin should be stable in the hydrophobic cavity provided by the CD dimer; thus, this force drives anion binding to iron(III)porphyrin–methylated CD complexes in water^43^. Hydrophobic H_2_S is easily accessible to the iron(III) center of met-hemoCD, followed by deprotonation to SH^–^ to neutralize the net charge of iron(III)porphyrin. The pH-dependent binding character is explained by the state of hydrogen sulfide (H_2_S or SH^–^) and the axial ligand species on the iron(III) center before binding. Met-hemoCD-I has an exchangeable aqua ligand at neutral pH due to its p*K*_a_^H2O^ (7.7), whereas met-hemoCD-P (p*K*_a_^H2O^ = 5.5) has a strongly coordinated hydroxo ligand on iron(III) at neutral pH. Therefore, met-hemoCD-I, which exhibits a higher p*K*_a_^H2O^ than that of met-hemoCD-P, bound more quickly to hydrogen sulfide under physiological conditions; thus, met-hemoCD-I could be superior to met-hemoCD-P as a hydrogen sulfide receptor in vivo.

The interaction between hydrogen sulfide and synthetic iron(III) porphyrins has been investigated through biomimetic chemistry. In the system that involves a picket-fence-type Fe/Cu binuclear porphyrin complex, electrocatalytic O_2_ reduction on the gold electrode was inhibited at a high concentration of H_2_S in a reversible manner^44^. This result suggests the toxic mechanism by which H_2_S inhibits the native mitochondrial C*c*O system. Another study using picket-fence porphyrins in nonaqueous media revealed that stable HS–Fe(III)porphyrin complexes formed via a 1:1 reaction with the SH^–^ anion^45^. A synthetic heme-peptide conjugate model was also synthesized as an MP-11 model and formed a 6-coordinated HS–Fe(III) low-spin species in water^46^. Atmospheric oxygen causes the porphyrins to significantly decompose, which is common in these model studies; thus, these HS–Fe(III)porphyrin complexes have been characterized under anaerobic conditions. Due to the difficulty in preparing HS–heme species, a stable synthetic model with the R_3_Si–S–Fe(III) complex has been proposed for detailed structural characterization^47^. To our knowledge, in presence of oxygen, there are few artificial synthetic models for the binding between hydrogen sulfide and iron-porphyrins in water except for our previous study using the hemoCD system^36^. Importantly, in our model, once hydrogen sulfide bound to the met-hemoCDs, the porphyrin ring was significantly protected against oxidative degradation induced by SH^–^ owing to the CD cavity and axial fifth coordination. Due to its high stability, the bound SH^–^ anion could be efficiently converted to sulfite and sulfate ions via oxidation by atmospheric O_2_. This oxidative conversion from hydrogen sulfide to sulfite, sulfate and thiosulfate ions or polysulfides has been similarly reported in the native Hb and Mb systems^35,37,48^, in which the heme cofactors are also protected in the hydrophobic heme pockets provided by apo-proteins. Therefore, due to the protection of the porphyrin ring by the CD dimer, we efficiently detoxified hydrogen sulfide under physiological conditions. Furthermore, in contrast to Mb and Hb, injected met-hemoCD is easily excreted in the urine due to its small molecular weight, which is another advantage of the present antidote system.

Currently, no antidote is available to clinically treat H_2_S poisoning, but several approaches have been proposed to detoxify hydrogen sulfide in animals and clinical trials. As the toxic mechanism is almost identical to that of hydrogen cyanide, vitamin B_12_ analogs (hydroxocobalamin and cobinamide), which are used as cyanide antidotes, are effective treatments for hydrogen sulfide poisoning^4,17,49,50^. Intravenously injected vitamin B_12_ analogs can capture hydrogen sulfide during circulation, after which it is strongly captured by serum proteins and accumulates for a long period (over a month)^24,51^. Therefore, patients that receive high doses of vitamin B_12_ analogs may need to avoid strong light exposure due to the photosensitizing property of cobalamins. Oxidized Hb (met-Hb) could function as an antidote for hydrogen sulfide. Sodium nitrite (NaNO_2_), which is an oxidizing agent that produces met-Hb in circulating RBCs, has been shown to exhibit an antidote effect on hydrogen sulfide^52^. However, this method cannot be easily adjusted to the met-Hb ratio (%) in RBCs. In another recent trial using met-Hb, a met-Hb-albumin cluster was injected and markedly improved the survival rate of mice^18^. Compared to these potential candidates, the hemoCD system is advantageous because (1) met-hemoCD-I shows greater binding affinity toward hydrogen sulfide than that of met-Hb and met-Mb, and (2) the hemoCD compound injected is quantitatively excreted in urine within several hours. Therefore, met-hemoCD-I can effectively capture H_2_S, and after detoxification, injected met-hemoCD-I easily disappears from the body through renal clearance. In a previous study, ferrous hemoCD-P injected into mice or rats was quantitatively detected in the CO-bound form in urine^21,23,25^. However, for hydrogen sulfide, HS-bound met-hemoCD-I was not detected in the urine. Instead, met-hemoCD-I was mainly detected without change because the bound hydrogen sulfide on met-hemoCD-I in the body could be smoothly converted to sulfite or sulfate ions via catalytic oxidation with O_2_, as demonstrated in this study.

We recently reported an antidote system for CO and cyanide mixed intoxication using hemoCD-Twins, a mixture of hemoCD-P and hemoCD-I in the ferrous state^25^. It is well known that CO and HCN are generated as lethal toxic gases during building fires. Previous studies in mice demonstrated that CO and HCN exhibit a synergetic lethal effect, which was counteracted by hemoCD-Twins. Interestingly, when hemoCD-Twins was injected into mice, hemoCD-P remained ferrous and could capture CO, while hemoCD-I was oxidized during circulation, and met-hemoCD-I detoxified cyanide. In general, CO poisoning can be quickly diagnosed by pulse CO oximetry or blood gas analysis. On the other hand, HCN in blood is difficult to detect rapidly at the site of accidents. The dual antidote system with hemoCD-Twins is advantageous because it can be injected without determination of which gas the patient primarily inhaled. Additionally, in this study, met-hemoCD-I clearly detoxified hydrogen sulfide. Although hydrogen sulfide is rarely produced in fire accidents, hemoCD-Twins are the first choice for patients that are potentially poisoned by an unknown gas. As the injected hemoCD complex is smoothly excreted in urine, it can be employed without a risk of side effects due to accumulated compounds. We are attempting to apply this detoxification system using hemoCD-Twins in clinical practice.

In conclusion, synthetic heme model compounds composed of iron(III)porphyrin and a per-*O*-methylated cyclodextrin dimer were found to bind to hydrogen sulfide and form a stable HS–Fe(III) complex. Furthermore, met-hemoCD-I catalytically and efficiently decomposed hydrogen sulfide into nontoxic sulfite/sulfate ions under physiological conditions. Mouse animal experiments revealed that met-hemoCD-I exhibits excellent properties as a novel antidote for hydrogen sulfide poisoning. We expect that this hemoCD-based system will serve as a ready-to-use, multifunctional gas poisoning antidote that can simultaneously remove CO, cyanide, and hydrogen sulfide via a single injection.

## Materials and methods

### Materials

 Met-HemoCD-P and met-hemoCD-I were synthesized in our laboratory as described in previous reports^25^. We used sodium hydrogen sulfide (NaSH) as a hydrogen sulfide source. NaSH was purchased from Stream Chem. Inc. Before the use of NaSH, its purity was determined by iodometric titration^36^. All other reagents were purchased and used as received. The solution was deoxidized by N_2_-bubbling for 30 min prior to anaerobic experiments.

### Preparation of the NaSH solution

 Since NaSH is easily oxidized in water, we prepared a stock solution of NaSH before each application. Milli-Q water was deoxidized by nitrogen gas bubbling for 30 min. Then, NaSH was solubilized in deoxidized water and used as a stock solution. Once a NaSH solution was prepared, it was used within 3 h. The concentration of NaSH in solution, *C* [mM], was determined as follows:

$$\:C\:\left[\text{m}\text{M}\right]=\left\{\left(x\:\left[\text{m}\text{g}\right]\times\:\:y\left[\%\right]\times\:{10}^{3}\right)/\left(56.063\left[\text{g}\cdot\:{\text{m}\text{o}\text{l}}^{-1}\right]\times\:z\left[\text{m}\text{L}\right]\right)\right\}$$

where *x* is the weight of NaSH, *y* is the purity of NaSH determined by iodometric titration as described above, and *z* is the volume of the stock solution. The purity of NaSH was 93%.

### Instruments

 UV‒vis spectra were recorded on a Shimadzu UV-2450 spectrophotometer with a thermostatic cell holder. Kinetic studies were carried out using a JASCO FS-110 Fast Scan Spectrometer with a thermostatic cell holder. EPR measurements were obtained with a Bruker E500 spectrometer at the Institute for Molecular Science in continuous-wave (CW) mode operating at ~ 9.66 GHz and equipped with an Oxford Instruments ESR900 continuous helium flow cryostat. The experimental parameters were 5 mW microwave power, 100 kHz field modulation, and 10 G modulation amplitude. MALDI-TOF mass spectra were measured on Bruker Daltonics Autoflex speed spectrometers in positive mode. α-Cyano-4-hydroxycinnamic acid (CHCA) was used as the matrix. ESI-TOF mass spectra were taken on a JEOL JMS-T100CS spectrometer.

### Spectroscopic measurements

 The binding constants (*K*) of hydrogen sulfide to met-hemoCD-P and met-hemoCD-I were determined by UV‒vis photometric titration. NaSH (up to 50 µM) was added to an aerobic solution of met-hemoCD-I or met-hemoCD-P (5 µM) at 25 °C in 0.05 M phosphate buffer at pH 6.0, 7.0, 7.4 and 8.0. The absorbance changes at the wavelength with maximum change in differential spectrum versus [NaSH] were plotted, and the data were fitted to a theoretical curve of an equation for 1:1 complex formation to obtain *K*.

The association rate constants (*k*_on_) for the binding of hydrogen sulfide to met-hemoCD-P and met-hemoCD-I were obtained under pseudo-first-order conditions with excess NaSH at 25 °C. An aerobic solution of met-hemoCD-P or met-hemoCD-I (5 µM) was mixed rapidly with various concentrations of NaSH (100–1400 µM before mixing) in 0.05 M phosphate buffer at pH 7.0, 7.4 or 8.0. The change in absorbance at 410 nm was monitored, and the data were fitted to a single or double exponential function to obtain the observed rates *k*_obs_. When the reaction between met-hemoCD and hydrogen sulfide was too fast (*k*_obs_ > 0.5), a double exponential fitting was required due to an unknown slow reaction component. In that case, the component of *k*_fast_ occupied more than 80% of the total. In addition, that ratio was independent of pH. Therefore, we adopted *k*_fast_ as the actual *k*_obs_ (reaction between met-hemoCDs and hydrogen sulfide), while *k*_slow_ is possibly due to the reaction between met-hemoCDs and impurities derived from NaSH, such as polysulfides^53^. Finally, the *k*_on_ values were obtained from linear regression of *k*_obs_ as a function of the hydrogen sulfide concentration. Concerning the binding parameters, we determined *k*_off_ from *K* and *k*_on_ (*K* = *k*_on_/*k*_off_). On the other hand, it should also be possible to determine *k*_off_ from y-intercept of the *k*_obs_ vs. [NaSH] plot (*k*_obs_ = *k*_on_ [NaSH] + *k*_off_). However, the estimated *k*_off_ values obtained from the plot seemed much larger than those determined based on *K* (Table 1). This is possibly due to the difference of the binding/dissociation species; as the *k*_on_ values became larger in lower pH, the binding species should be ascribed to undissociated H_2_S. Deprotonation after the binding results in the formation of HS–Fe(III) complexes. The *k*_off_ value based on *K* should be the dissociation of SH^–^ from the HS-Fe(III), whereas *k*_off_ from the y-intercept of the *k*_obs_ plot is assumed to be the H_2_S–Fe(III) species. Therefore, the *k*_off_ values from the y-intercept became much larger than those based on *K*. However, we avoid discussing this point in detail because the value from y-intercept generally includes large error (over 100%).

### Quantification of hydrogen sulfide

 An aqueous solution of NaSH (1.0 mM) was mixed with met-hemoCD or met-Hb (50 µM) and incubated at 25 °C. The residual hydrogen sulfide concentrations were determined using the reported “methylene blue” method with some modifications^54,55^. The solution containing hydrogen sulfide and its scavenger was diluted to 2.5 mL with 0.05 M phosphate buffer at pH 7.4 to adjust the total hydrogen sulfide concentration to less than 50 µM. To the solution was added trifluoroacetic acid (0.5 mL). Then, solutions of *N*,* N*-dimethyl-*p*-phenylenediamine sulfate (200 mM stock solution, 30 µL) dissolved in HClaq (7.2 mM) and FeCl_3_ (300 mM stock solution, 30 µL) dissolved in 1.2 mM HCl were added successively. The solutions were allowed to stand for 20 min at ambient room temperature. Finally, the absorbance at 663 nm was read, and the hydrogen sulfide concentration was determined based on the standard curve of methylene blue.

### Quantification of sulfate and sulfite

 The relative production rate of sulfate and sulfite ions from hydrogen sulfide was determined by a simple turbidimetric method, in which the amount of sulfate (SO_4_^2–^) was quantified by turbidity through the following reaction:^56^.

$$SO_{4} ^{{2{-}}} + {\text{ }}BaCl_{2} \to {\text{ }}BaSO_{4} \downarrow {\text{ }} + {\text{ }}2Cl^{{-}}$$

The average absorbance between 700 nm and 800 nm was used as turbidity, where met-hemoCD-I and other scavengers showed no absorbance in the area. The turbidity showed a linear correlation with the sulfate concentration from 0.5 mM to 4.0 mM upon the addition of 1.5 equivalents of BaCl_2_. We also confirmed that sulfite (SO_3_^2–^) ions form a similar insoluble precipitate of BaSO_3_ with BaCl_2_ as follows:^57^.

$$SO_{3} ^{{2{-}}} + {\text{ }}BaCl_{2} \to {\text{ }}BaSO_{3} \downarrow {\text{ }} + {\text{ }}2Cl^{{-}} ~$$

Therefore, the relative production of sulfate and sulfite ions in solution was simply determined by reading the average absorbance between 700 nm and 800 nm after treatment of the solution with BaCl_2_.

### Animal experiments

 All animal studies were performed under the approval of Doshisha University and the animal experiments including all methods were carried out in accordance with the Guidelines for Animal Experiments of Doshisha University. We used female BALB/cCrSlc mice (Shimizu Laboratory Supplies, Co., Ltd.) weighing 20–22 g. The study is reported in accordance with ARRIVE guidelines and AVMA guidelines for the Euthanasia of Animals (2020). For acclimatization, the mice were housed under a 12 h/12 h light/dark cycle with free feedings under specific-pathogen-free (SPF) conditions for one week before the day in the experiments. The mice were euthanized using cervical dislocation method by a well-trained experimenter.

For survival analysis of met-hemoCD-I in lethal hydrogen sulfide intoxication model mice, a solution of NaSH (21 mg/kg) in PBS (0.1 mL) was injected intraperitoneally under unanesthesia. One minute after the injection of NaSH, a solution of met-hemoCD-I (7 mM) in PBS (0.2 mL) was injected intraperitoneally. For the predosing experiments, a solution of met-hemoCD-I (7 mM) in PBS (0.2 mL) was injected intraperitoneally. After 10 min, a solution of NaSH (21 mg/kg) in PBS (0.1 mL) was injected intraperitoneally. Survival rates were then monitored for one hour after hydrogen sulfide intoxication was induced.

We determined the C*c*O activity and the concentration of lactate in the blood of lethal hydrogen sulfide intoxication model mice. Tissue samples (brain, heart, and liver) were collected from met-hemoCD-I-treated surviving mice one hour after hydrogen sulfide intoxication was induced. As a control, we collected tissue samples (brain, heart, and liver) from untreated mice immediately after death. The CcO activity in these organs was determined by the following method according to the literature^58^. Each tissue sample (~ 20 mg) was homogenized in 0.5 mL of sucrose muscle homogenization buffer (250 mM). Then, the suspension was centrifuged at 600 g for 10 min at 4°C. The supernatant (5 µL) was added to a 1 mL cuvette which contains 400 µL of Milli-Q water, 500 µL of potassium phosphate buffer (0.1 M, pH 7.0) and 50 µL of reduced cytochrome *c* (Product name; ab109911, abcam, UK). The absorbance at 550 nm was read for 3 min. The rate of activity (OD/min) was determined by calculating the slope between two points within the linear region. Finally, the CcO activity was determined by normalizing the rate activity by its protein amount in tissue using BCA assay (Thermo Fisher Scientific, Japan). The concentration of lactate in the blood was measured using LT-1730 Lactate Pro2 (Arkray).

### Statistical analysis

 Statistical analyses were performed using GraphPad Prism, version 10.2.3 (GraphPad Software). All the data are presented as the means ± standard errors from at least three different experiments and were analyzed by Student’s t test. Survival curves were analyzed using Kaplan‒Meier curves and the log-rank test. Differences with *P* values less than 0.05 were considered significant.

## Electronic supplementary material

Below is the link to the electronic supplementary material.

Supplementary Material 1

## Data Availability

All relevant data are in the manuscript.
