# Supplementary material for: Detoxification of hydrogen sulfide by synthetic heme model compounds
Source: Sci Rep. 2024 Dec 10;14:29371. doi: 10.1038/s41598-024-80511-1 (PMC11632086; doi:10.1038/s41598-024-80511-1)
Supplement: Supplementary file 1 — Supplementary Material 1 [file 41598_2024_80511_MOESM1_ESM.docx]

***Figure S1.*** Typical UV-vis absorption spectra of various states of hemoCDs in 0.05 M phosphate buffer solution at pH 7.0 and 25°C. The strong absorption at wavelengths below 350 nm observed in deoxy-hemoCDs and CO-hemoCDs are due to the presence of Na_2_S_2_O_4_ as a reductant in the system.

***Figure S2.*** UV-vis absorption spectra of met-hemoCDs in 0.05 M phosphate buffer solution and 25°C and at different pH of the solution.

***Figure S3.*** UV-vis absorption spectral changes of (**A**) Fe(III)TPPS (5 μM) and (**B**) Fe(III)TPPS (5 μM) with TMe-β-CD (12 μM) in 0.05 M phosphate buffer solution at pH 7 upon addition of NaSH at 25^o^C. NaSH was stepwisely added to the solution at one minute interval, and the ratio of Fe(III)/NaSH was 1/3 at the end.

Inset: Plots of the absorbance changes versus [NaSH]. The spectral changes indicate degradation of the porphyrin ring by NaSH without specific interaction. Therefore, in case of met-hemoCD-P and met-hemoCD-I shown in Fig. 2 in the main text, axial fifth ligation is essential to form a stable HS–Fe(III) complex in aqueous solution.

******

***Figure S4.*** (a-c) UV‒vis absorption spectra collected over time in the reaction of met-hemoCD-P (5 μM) with excess NaSH in 0.05 M phosphate buffer solution at pH different pH and 25°C. The initial spectrum is shown in blue, and the final spectrum is shown in red. (d) The absorbance changes at 410 nm as a function of time upon addition of NaSH.

***Figure S5.*** (a-c) UV‒vis absorption spectra collected over time in the reaction of met-hemoCD-I (5 μM) with excess NaSH in 0.05 M phosphate buffer solution at pH different pH and 25°C. The initial spectrum is shown in blue, and the final spectrum is shown in red. (d) The absorbance changes at 410 nm as a function of time upon addition of NaSH.

***Figure S6.*** The continuous variation plot (Job’s plot) to determine the stoichiometric ratio for the reaction of met-hemoCD-I with hydrogen sulfide. (**A**) The raw UV-vis absorption spectra at various concentration of met-hemoCD-I without or with various molar ratio of NaSH. In the presence of NaSH, the total concentration of met-hemoCD-I and NaSH was adjusted to 10 μM. (**B**) Job’s plot based on the absorbance difference at 438 nm without and with NaSH. The maximum complexation ratio was observed at 1:1 molar ratio, indicating that met-hemoCD-I binds one SH^–^ anion on its iron(III) center in 0.05 M phosphate buffer solution at pH 7 at 25^o^C.

***Figure S7.*** Electrospray ionization time-of-flight (ESI-TOF) mass spectroscopic analysis for met-hemoCD-I without (**A**) and with NaSH (**B**). The aqueous solutions of these complexes were infused to the ionization chamber and the spectra were collected on negative mode. Insets show observed and simulated isotope distribution patterns of [met-hemoCD-I]^3–^ and [HS-hemoCD-I]^4–^. The MS analysis clearly indicates the structure of HS-coordinated ferric met-hemoCD-I with excluding the possibility of polysulfide-coordinated HS(S)_n_–Fe species.

***Figure S8.*** UV‒vis absorption spectral changes of met-hemoCD-I (5 μM) upon addition of two molar equivalents of NaSH in (a) aerobic and (b) anaerobic conditions in 0.05 M phosphate buffer solution at pH 7.4 and 25°C. This result indicates that the HS-complexes were similarly formed under aerobic and anaerobic conditions before starting homolytic reduction of iron and/or degradation of the iron porphyrin shown in Figure 5A.

***Figure S9.*** UV‒vis absorption spectral changes and its kinetic traces of Figure 5B, in which met-hemoCD-I (5 μM) was added to two molar equivalents of NaSH, and the CO gas was bubbled into the solution in 0.05 M phosphate buffer solution at pH 7.4 and 25°C.

***Figure S10.*** UV‒vis absorption spectral changes of met-hemoCD-I (5 μM) upon addition of two molar equivalents of NaSH, and the CO gas was bubbled into the solution (a) right after addition of NaSH, and (b) one hour addition of NaSH in 0.05 M phosphate buffer solution at pH 7.4 and 25°C.

***Figure S11.*** UV‒vis absorption spectral changes of oxy-hemoCD-I (5 μM) in the (a) absence or (b) presence of two molar equivalents of NaSH in 0.05 M phosphate buffer solution at pH 7.4 and 25°C. The initial spectrum is shown in blue, and the final spectrum is shown in red.

**Table 1.** Iodometric titration results of NaSH.

Weighed NaSH Amount of iodine Titrated 0.1 M Na_2_S_2_O_3_ Effective NaSH

*w* [mol] *y* [mol] *z* [mL] *x* [mol]

n : 1 5.04 × 10^-4^ 1.00× 10^-3^ 10.70 4.65 × 10^-4^

n : 2 5.04 × 10^-4^ 1.01× 10^-3^ 10.95 4.63 × 10^-4^

n : 3 5.04 × 10^-4^ 0.98× 10^-3^ 10.10 4.71 × 10^-4^

Ave. 5.04 × 10^-4^ 4.66 × 10^-4^

The effective NaSH was calculated as follows.

*x* [mol] = (2 – *z*×(0.1/1000))/2

Therefore, the purity of NaSH was determined by *x*/*w*, which provided 92.5%.
